# Das RECORD-Statement zum Berichten von Beobachtungsstudien, die routinemäßig gesammelte Gesundheitsdaten verwenden

**DOI:** 10.1016/j.zefq.2016.07.010

**Published:** 2016-10

**Authors:** Eric I. Benchimol, Liam Smeeth, Astrid Guttmann, Katie Harron, Lars G. Hemkens, David Moher, Irene Petersen, Henrik T. Sørensen, Erik von Elm, Sinéad M. Langan, RECORD Working Committee

**Affiliations:** a Children's Hospital of Eastern Ontario Research Institute, Department of Pediatrics and School of Epidemiology, Public Health and Preventive Medicine, University of Ottawa, Ottawa, Canada; b Institute for Clinical Evaluative Sciences, Toronto, Canada; c London School of Hygiene and Tropical Medicine, London, United Kingdom; d Hospital for Sick Children, Department of Paediatrics and Institute of Health Policy, Management and Evaluation, University of Toronto, Toronto, Canada; e Basel Institute for Clinical Epidemiology and Biostatistics, University Hospital Basel, Switzerland; f Ottawa Hospital Research Institute, Ottawa, Canada, and School of Epidemiology, Public Health and Preventative Medicine, University of Ottawa, Ottawa, Canada; g Department of Primary Care and Population Health, University College London (UCL), London, United Kingdom; h Department of Clinical Epidemiology, Aarhus University, Aarhus, Denmark; i Cochrane Switzerland, Institute of Social and Preventive Medicine, University Medical Centre Lausanne, Lausanne, Switzerland

**Keywords:** Leitlinien für Forschungsberichte, Beobachtungsstudien, Epidemiologische Methoden, Konsensus, Gesundheitsbezogene Routinedaten, Administrative Gesundheitsdaten, Elektronische Gesundheitsdaten, Datenbanken zur Grundversorgung, Dokumentation, Informationsverbreitung, Fall-Kontroll-Studien, Kohortenstudien, Wissensverbreitung, Medizinischen Zeitschriften, Wissenschaftliche Zeitschriften, Zeitschriften mit Peer-Review, Publizieren, reporting guidelines, observational research, epidemiologic methods, consensus, routinely collected health data, health administrative data, electronic health data, documentation, information dissemination, case-control studies, cohort studies, knowledge dissemination, medical journals, scientific journals, publishing, CPRD, Clinical Practice Research Datalink, GPRD, General Practice Research Database, HSMR, Hospital Standardised Mortality Ratio, ICD, International Classification of Diseases, ISC, New South Wales Inpatient Statistics Collection, MDC, New South Wales Midwives Data Collection, MeSH, Medical Subject Heading, mHealth-Apps, mobile Gesundheitsapplikationen, NHS, National Health Service, NSCLC, Non-Small Cell Lung Cancer, PET, Positronen-Emissions-Tomographie, PICANet, Paediatric Intensive Care Audit Network, RECORD, REporting of studies Conducted using Observational Routinely collected health Data, SEER, Surveillance, Epidemiology, and End Results, SNIIRAM, Système National d’Informations Inter Régimes de l’Assurance Maladie, STROBE, Strengthening the Reporting of Observational Studies in Epidemiology

## Abstract

Zunehmend werden routinemäßig gesammelte Gesundheitsdaten, die zu administrativen und klinischen Zwecken und ohne spezifische, a priori festgelegte Forschungsziele erhoben wurden, auch für die Forschung eingesetzt. Die rasche Entwicklung und Verfügbarkeit dieser Daten machten Probleme deutlich, die in den bestehenden Berichts-Leitlinien, wie dem STROBE-Statement (Strengthening the Reporting of Observational Studies in Epidemiology) nicht behandelt werden. Das RECORD-Statement (REporting of studies Conducted using Observational Routinely-collected health Data) wurde entwickelt, um diese Lücken zu schließen. RECORD ist als Erweiterung des STROBE-Statements gedacht, um Punkte abzudecken, die spezifisch sind beim Berichten von Beobachtungsstudien, die routinemäßig gesammelte Gesundheitsdaten verwenden. RECORD besteht aus einer Checkliste von 13 Punkten mit Bezug zu Titel, Abstract, Einleitung, Methoden-, Ergebnis- und Diskussionsteil von Artikeln sowie zu anderen Informationen, die in Forschungsberichten dieser Art enthalten sein sollten. Dieses Dokument enthält die Checkliste sowie Erläuterungen und weitere Erklärungen, um die Verwendung der Checkliste zu verbessern. Beispiele für ein gutes Berichten der einzelnen Punkte der RECORD-Checkliste sind ebenfalls in diesem Dokument enthalten. Dieses Dokument sowie die zugehörige Website und ein Forum (http://www.record-statement.org) werden die Umsetzung und das Verständnis von RECORD verbessern. Autoren, Redakteure von Fachzeitschriften und Peer-Reviewer können die Transparenz beim Berichten von Forschungsergebnissen erhöhen, indem sie RECORD anwenden.

## Abstract (en)

Routinely collected health data, obtained for administrative and clinical purposes without specific *a priori* research goals, are increasingly used for research. The rapid evolution and availability of these data have revealed issues not addressed by existing reporting guidelines, such as Strengthening the Reporting of Observational Studies in Epidemiology (STROBE). The REporting of studies Conducted using Observational Routinely collected health Data (RECORD) statement was created to fill these gaps.

RECORD was created as an extension to the STROBE statement to address reporting items specific to observational studies using routinely collected health data. RECORD consists of a checklist of 13 items related to the title, abstract, introduction, methods, results, and discussion section of articles, and other information required for inclusion in such research reports. This document contains the checklist as well as explanatory and elaboration information to enhance the use of the checklist. Examples of good reporting for each RECORD checklist item are also included. This document, as well as the accompanying website and message board (http://www.record-statement.org), will improve the implementation and understanding of RECORD. By implementing RECORD, authors, journals editors, and peer reviewers can enhance transparency of research reporting.

## Einführung

In der Gesundheitsversorgung und beim Monitoring von Krankheitsentstehung und -entwicklung werden mehr und mehr Daten verfügbar. Dies hat die Forschungslandschaft verändert. Routinemäßig gesammelte Gesundheitsdaten (im weiteren kurz „Routinedaten“ genannt) können als Daten definiert werden, die gesammelt werden, ohne dass vor ihrer Verwendung zu Forschungszwecken eine spezifische, a priori festgelegte Forschungsfrage entwickelt wurde [1]. Solche Daten können von einem breiten Spektrum von Ressourcen stammen im Bereich der Forschung (z. B. Krankheitsregister), des klinischen Managements (z. B*.* Datenbanken zur Grundversorgung), der Planung des Gesundheitssystems (z. B*.* Verwaltungsdaten), Dokumentation der klinischen Versorgung (z. B. Archive von elektronische Patientenakten) oder zur epidemiologischen Überwachung (z. B*.* Krebsregister und Daten der Gesundheitsberichterstattung) gehören. Diese Daten, erhoben in verschiedenen medizinischen Versorgungsbereichen und geografischen Regionen, bieten Möglichkeiten zur innovativen, effizienten und kostenwirksamen Forschung. Sie fließen ein in Entscheidungen in der klinischen Medizin, in der Planung von Gesundheitsdiensten und in Public Health [2]. Auf internationaler Ebene haben sowohl Regierungen als auch Förderinstitutionen der Nutzung von Routinedaten als Mittel zur Verbesserung der Patientenversorgung, Umgestaltung der Gesundheitsforschung und Steigerung der Effizienz in der Gesundheitsversorgung Priorität eingeräumt [3].

Während die explosionsartige Zunahme verfügbarer Daten die Möglichkeiten zur Beantwortung drängender Forschungsfragen erheblich verbessert, stellt sie diejenigen, die Forschung selbst durchführen oder sie bewerten sowie für die Umsetzung der Forschungsergebnisse zuständig sind, vor Herausforderungen. Aufgrund des breiten Spektrums der Quellen, aus denen Routinedaten stammen, und der rasanten Erweiterung dieses Feldes, ist es schwierig, die Stärken und Schwächen einzelner Datenquellen sowie mit ihnen einhergehenden Bias (systematische Fehler) zu erkennen. Dies wird durch ein unvollständiges oder inadäquates Berichten von Forschung auf Basis von Routinedaten noch weiter erschwert. Eine systematische Analyse einer Stichprobe von Studien, in denen Routinedaten verwendet wurden, fand in den verschiedensten Bereichen unvollständiges oder unklares Berichten [4]. Zu den Mängeln in der Berichterstattung gehören inadäquate oder fehlende Informationen zur Codierung von Expositionen bzw. Zielgrößen und zum Anteil an verknüpften Datensätzen („linkage rates“) aus den verschiedenen Datenquellen. Zwei jüngst erschienene systematische Reviews dokumentieren ebenfalls, dass Studien zur Validierung von Daten aus Routinedatenquellen schlecht berichtet werden [5], [6]. Dies kann dazu führen, dass Ursachen von Bias nicht erkannt, Bemühungen zur Durchführung von Metaanalysen behindert und falsche Schlussfolgerungen gezogen werden.

### Feld 1. Definitionen der Begriffe Quell-, Datenbank- und Studienpopulation

**Abbildung 1:**
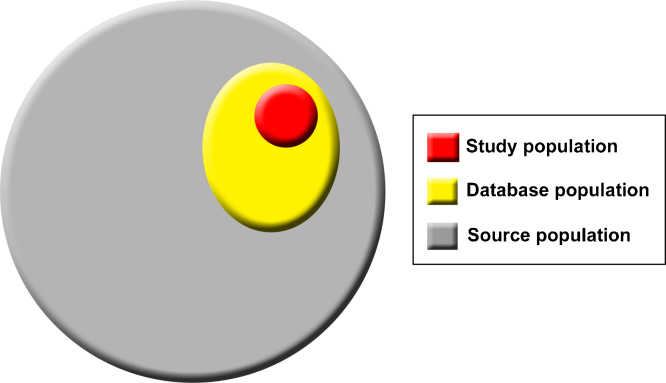
Hierarchie von Bevölkerungsgruppen in Studien, die Routinedatenquellen verwenden.

Für Studien, die Routinedaten verwenden, gibt es drei relevante Hierarchieebenen der Bevölkerungsgruppen, auf die in diesem Statement wiederholt Bezug genommen wird: Zu diesen Gruppen gehören (1) die Quellpopulation, worunter die Bevölkerungsgruppe verstanden wird, aus der sich die Datenbankpopulation ableitet und über die Forscher eine Aussage treffen möchten, (2) die Datenbankpopulation, die sich von der Quellpopulation ableitet und Personen mit Angaben in der Datenquelle umfasst, und (3) die Studienpopulation, die von den Forschern mit Hilfe von Codes und Algorithmen aus der Datenbankpopulation heraus definiert wird (Abb. 1) [7]. Beispielhaft sei hier das Projekt „Clinical Practice Research Datalink (CPRD)“ genannt, bei dem die Quellpopulation alle Personen umfasst, die im Vereinigten Königreich bei einem Allgemeinmediziner vorstellig werden. Die Datenbankpopulation umfasst diejenigen Personen, die in CPRD erfasst sind, und die Studienpopulation solche, die mit Hilfe von Codes und Algorithmen, die in der jeweiligen Studie beschrieben sind, aus CPRD ausgewählt wurden.

Als Hilfestellung für die Berichterstattung wurden für eine Reihe von Studiendesigns und Kontexte Leitlinien entwickelt. Sie gehen oft mit einer verbesserten Qualität von Studienberichten einher [8], [9]. Das *Strengthening the Reporting of Observational Studies in Epidemiology* (STROBE)-Statement wurde entwickelt, um die Transparenz beim Berichten von Beobachtungsstudien zu erhöhen [10], [11]. Es wird von führenden medizinischen Fachzeitschriften anerkannt und angewendet und verbessert bei Einbindung in den Redaktionsprozess nachweislich die Berichtsqualität von Forschungsergebnissen [12], [13]. Studien, die Routinedaten verwenden, haben meistens ein beobachtendes Design, weshalb die STROBE-Leitlinien hier relevant und anwendbar sind. Da das STROBE-Statement jedoch für alle Arten von Beobachtungsstudien ausgelegt ist, werden spezielle Aspekte von Forschung mit Routinedaten nicht berücksichtigt. Im Anschluss an das Primary Care Database Symposium in London im Jahr 2012 hat sich eine Gruppe internationaler Wissenschaftler, die sich insbesondere mit der Verwendung von Routinedaten beschäftigen, mit Vertretern der STROBE-Gruppe getroffen, um STROBE im Zusammenhang mit Studien, die Routinedaten verwenden, zu diskutieren [14], [15]. Dabei wurden in STROBE Lücken in Bezug auf die Forschung mit besagten Datenquellen identifiziert, und man ist übereingekommen, dass eine Erweiterung von STROBE vonnöten ist. So wurde die *REporting of studies Conducted using Observational Routinely collected Data* (RECORD)-Initiative als internationale Zusammenarbeit zur Erweiterung von STROBE ins Leben gerufen, um spezielle Berichtsprobleme im Zusammenhang mit Studien, die Routinedaten verwenden, zu erörtern und anzugehen. An der RECORD-Initiative waren bisher mehr als 100 internationale Stakeholder beteiligt, darunter Forscher, Redakteure von Fachzeitschriften und Nutzer von Daten, einschließlich solcher, die auf Routinedaten basierende Forschungsergebnisse in der Entscheidungsfindung gebrauchen. Die Methodik, die zur Entwicklung der RECORD-Leitlinien verwendet wurde, wird anderweitig ausführlich beschrieben [16] und basiert auf etablierten Verfahren zur Entwicklung von Leitlinien zur Berichterstattung [17]. Kurz beschrieben, wurden hierfür die Stakeholder zwei Mal befragt, um Themen für das RECORD-Statement festzulegen und zu priorisieren. Im Anschluss traf sich das Working Committee zur Ausarbeitung des Wortlauts der einzelnen Empfehlungen. Dann lasen die Stakeholder die Texte und gaben Rückmeldungen. Die Endversion der Checkliste und der vorliegende erläuternde Artikel wurden (im englischen Original) von Mitgliedern des Steering Committees ausgearbeitet und vom Working Committee überprüft und genehmigt. Mitglieder des STROBE-Leitungsgruppe waren an der Erstellung von RECORD beteiligt.

Wie schon bei STROBE, sind auch die RECORD-Leitlinien nicht darauf ausgelegt, Methoden zur Durchführung von Forschung zu empfehlen, sondern sie sollen vielmehr das Berichten über eben diese Forschung verbessern. So soll sichergestellt werden, dass Leser, Peer-Reviewer, Redakteure von Fachzeitschriften und andere Nutzer der Forschung die interne und externe Validität der Studien beurteilen können. Durch eine Verbesserung der Berichtsqualität von Forschung mit Routinedaten wollen wir die Anzahl unklarer Forschungsberichte verringern und so den Grundsätzen des wissenschaftlichen Prozesses gerecht zu werden: Entdeckung, Transparenz und Nachvollziehbarkeit [18].

## Punkte der RECORD-Checkliste

Tabelle 1 enthält die vollständige RECORD-Checkliste. Da RECORD eine Erweiterung der bereits vorhandenen STROBE-Punkte ist, werden die einzelnen Empfehlungen, nach den Abschnitten eines Manuskripts geordnet, neben den entsprechenden Punkten der STROBE-Checkliste aufgeführt. Wir empfehlen Autoren, auf jeden Punkt der Checkliste hinreichend einzugehen, aber schreiben keine präzise Reihenfolge oder Position im Manuskript vor. Sortiert nach Manuskriptabschnitt geben wir nachstehend für jeden Punkt der RECORD-Checkliste detaillierte Erklärungen. Wenn kein weiterer Checklisten-Punkt benötigt wurde, um STROBE auch auf Studien mit Routinedaten anwenden zu können, finden sich eventuell eine Erklärung unter dem entsprechenden STROBE-Punkt.

### Titel und Abstract

RECORD-PUNKT 1.1: Der verwendete Datentyp sollte im Titel oder Abstract angegeben werden. Die Namen der verwendeten Datenbanken sollten, sofern möglich, aufgeführt werden. RECORD-PUNKT 1.2: Gegebenenfalls sollte die geografische Region und der Zeitrahmen, in dem die Studie durchgeführt wurde, im Titel oder Abstract angegeben werden.

RECORD-PUNKT 1.3: Wurde für die Studie eine Verknüpfung von Datenbanken vorgenommen, sollte dies klar im Titel oder Abstract angegeben werden.

#### Beispiele

Zu diesen Punkten sind zwei Beispiele für gutes Berichten in den folgenden Artikeln enthalten:

1. „Perforations and Haemorrhages after Colonoscopy in 2010: A Study Based on Comprehensive French Health Insurance Data (SNIIRAM)“ [19].
2. „The Dutch Hospital Standardised Mortality Ratio (HSMR) Method and Cardiac Surgery: Benchmarking in a National Cohort Using Hospital Administration Data versus a Clinical Database“ [20].

#### Erläuterung

Da es kein akzeptiertes Stichwort im Medical Subject Heading (MeSH) Thesaurus gibt, um Studien, die Routinedaten verwenden, zu erkennen, ist es wichtig, dass eine Studie, die mit Hilfe dieser Daten durchgeführt wird, als solche identifiziert werden kann. Jedoch reicht es angesichts der Vielzahl unterschiedlicher Datentypen nicht aus, lediglich anzugeben, dass Routinedaten verwendet wurden. Stattdessen sollte der verwendete Typ von Routinedaten im Titel oder Abstract angegeben werden. Dies können etwa gesundheitsbezogene Verwaltungsdaten, andere Verwaltungsdaten (z. B. aus Versicherungen, Geburts-/Sterberegister oder von Arbeitgebern), Krankheitsregister, Datenbanken der Grundversorgung, elektronische Patientenakten und bevölkerungsbasierte Register, sein. Es ist wichtig, den Namen der verwendeten Datenbank(en) anzugeben; dies sollte aber nicht die Angabe des Typs der Datenquelle im Titel oder Abstract ersetzen.

Die geografische Region und der Zeitrahmen sind Punkte in der STROBE-Checkliste. Wir schlagen vor, dass diese Information ebenfalls ein erforderlicher Punkt sein sollte im Titel oder Abstract von Manuskripten, welche die RECORD-Checkliste verwenden. Dabei müssen sich Angaben zu Ort und Zeitrahmen natürlich an der zugelassenen Wörterzahl orientieren. Zudem gilt es, die Vertraulichkeit von Informationen zu wahren. Dennoch sollte der Ort zumindest auf der übergeordneten geografischen Ebene berichtet werden, die zur Definition der Studienpopulation verwendet wurde (z. B. Staat, Bundesland, Provinz oder Region).

Des Weiteren sollten Verknüpfungen zwischen Datenbanken (sofern diese vorgenommen wurden) im Titel oder Abstract angegeben werden. Ein geeigneter Wortlaut ist zum Beispiel „unter Verwendung mehrerer verknüpfter administrativer Datenbanken“ oder „(Name der Datenbank) verknüpft mit (Name der Datenbank)“. Die Verwendung des Wortes „verknüpft“ (linked) oder „Verknüpfung“ (linkage) sind im Titel oder Abstract ausreichend. Weitere Einzelheiten zur Verknüpfungsmethode sollten im Methodenteil des Manuskripts angegeben werden.

### Einleitung

Neben den STROBE-Punkten sind keine für die RECORD-Leitlinien spezifischen Punkte erforderlich. Gemäß den STROBE-Leitlinien sind „alle spezifischen Zielsetzungen einschließlich der (vorab festgelegten) Hypothesen“ in der Einleitung anzugeben. Für die Replikation und Translation von Beobachtungsstudien ist die Angabe der spezifischen Forschungsziele unerlässlich. Bei Studien, die Routinedaten verwenden, sollten die Autoren ferner präzisieren, ob die Analysen explorativer Natur sind, d.h. zum Aufdecken neuer Beziehungen anhand der Daten (Beispiele: „Data mining“-Studien oder hypothesengenerierende Studien [21], [22]) oder konfirmatorischer Natur d.h. zur Prüfung einer oder mehrerer Hypothesen [23]. Die Autoren sollten überdies angeben, ob ihre Hypothesen vor oder nach der Datenanalyse aufgestellt wurden. Sie sollten eindeutige Angaben darüber machen, ob es ein Studienprotokoll gibt und wie auf dieses zugegriffen werden kann, und ob die Studie in einem öffentlich zugänglichen Studienregister eingetragen ist. Da die Stärken und Schwächen der in der Forschung mit Routinedaten verwendeten Methoden umstritten sein könnten, ist eine klare Beschreibung der Studienziele unerlässlich [23], [24]. Es ist nicht ausreichend, eine Studie lediglich als deskriptiv zu bezeichnen, ohne klarzustellen, ob die Studie darauf ausgelegt ist, Hypothesen zu generieren oder zu prüfen.

### Methoden (Rahmenbedingungen)

Es sind keine weiteren RECORD-Punkte erforderlich zur Erweiterung der in STROBE enthaltenden folgenden Anforderung: „Beschreiben Sie den Rahmen (Setting) und Ort der Studie und machen Sie relevante zeitliche Angaben, einschließlich der Zeiträume der Rekrutierung, der Exposition, der Nachbeobachtung und der Datensammlung“. Autoren sollten beachten, dass zusätzlich zum Typ der Datenbank, der bereits im Titel und/oder Abstract erwähnt wurde, weitere Informationen bereitgestellt werden sollten, damit der Leser den Inhalt und die Validität der Datenbank sowie die ursprünglichen Gründe für die Datenerfassung nachvollziehen kann. So kann beispielsweise eine elektronische Patientenakte von Spezialisten oder Grundversorgern, zur ambulanten oder stationären Versorgung, oder von erfahrenen Ärzten oder Medizinstudenten verwendet werden. Die Benutzer werden möglicherweise gezielt für eine gründliche und reproduzierbare Dateneingabe geschult oder führen diese ohne Schulung aus [25]. Autoren sollten auch beschreiben, in welchem Zusammenhang die Datenbankpopulation mit der Quellpopulation steht, einschließlich der Auswahlkriterien, damit die Leser nachvollziehen können, ob die Ergebnisse auf die Quellpopulation angewandt werden können.

### Methoden (Teilnehmer)

RECORD-PUNKT 6.1: Die Methoden für die Auswahl der Studienpopulation (wie verwendete Codes oder Algorithmen zur Identifizierung von Teilnehmern) sollten detailliert aufgelistet werden. Ist dies nicht möglich, sollte dies erklärt werden.

RECORD-PUNKT 6.2: Für alle Studien zur Validierung der Codes und Algorithmen, die für die Auswahl von Teilnehmern verwendet wurden, sollten Referenzen angegeben werden. Wurde eine Validierung für diese Studie durchgeführt und nicht anderweitig veröffentlicht, sollten die Methoden und Ergebnisse detailliert dargestellt werden.

RECORD-PUNKT 6.3: Wurden in der Studie Datenbanken verknüpft, sollte zur Darstellung des Verknüpfungsprozesses die Verwendung eines Flussdiagramms oder einer anderen grafischen Darstellung in Betracht gezogen werden. Dies sollte auch die Anzahl von Personen mit verknüpften Daten in den einzelnen Abschnitten enthalten.

#### Beispiele

RECORD-PUNKT 6.1: Der folgende Auszug ist ein Beispiel für gutes Berichten:

Die OCCC [Ontario Crohn's and Colitis Cohort] verwendet validierte Algorithmen zur Identifizierung von Patienten mit CED (chronisch entzündliche Darmerkrankung) nach Altersgruppe. Jeder dieser Algorithmen wurde in Ontario in der jeweiligen Altersgruppe, auf die er angewandt wurde, in mehreren Kohorten, Arten von Arztpraxen und Regionen validiert. Bei Kindern unter 18 Jahren wurde der Algorithmus je nachdem, ob sich diese einer Koloskopie oder einer Sigmoidoskopie unterzogen, definiert. Für Kinder mit einer Endoskopie waren 4 ambulante Arztkontakte oder 2 CED-bedingte Hospitalisierungen innerhalb von 3 Jahren nötig. Bei Kindern, die keine Endoskopie hatten, waren 7 ambulante Arztkontakte oder 3 CED-bedingte Hospitalisierungen innerhalb von 3 Jahren nötig.… Dieser Algorithmus identifizierte Kinder mit CED richtig mit einer Sensitivität von… [26].

Dieser Artikel referenziert zwei frühere Validierungsstudien von Algorithmen zur Identifizierung von Patienten mit entzündlicher Darmerkrankung unterschiedlichen Alters einschließlich Messgrößen zur diagnostischen Güte.  

**Abbildung 2:**
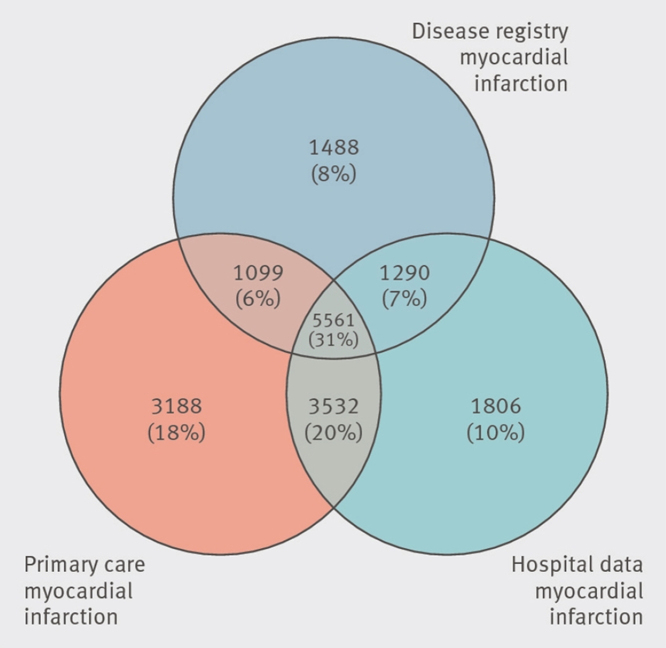
Venn-Diagramm zur Darstellung des Verknüpfungsprozesses (reproduziert mit Erlaubnis von Herrett et al. [28] auf unserer Website: http://record-statement.org/images/figure2.jpg).

RECORD-PUNKT 6.2: 1. In ihrem Artikel beschrieben Ducharme et al. ausführlich die Validierung der Codes zur Identifizierung von Kindern mit Darminvagination und verwendeten anschließend die validierten Codes zur Beschreibung der Epidemiologie. Die Codes der Validierungsstudie sind in Abbildung 2 des Artikels aufgeführt [27].

2. In ihrem Artikel nannten Benchimol et al. keine Validierung, jedoch wurden bislang durchgeführte Validierungen referenziert. Details der diagnostischen Güte der Codes des Identifizierungsalgorithmus wurden beschrieben [26].

**Abbildung 3:**
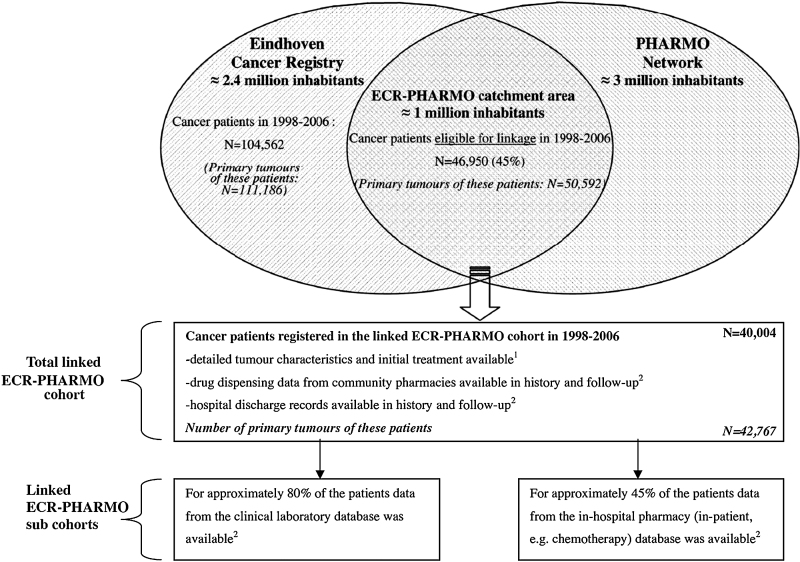
Kombination aus Flussdiagramm und Venn-Diagramm zur Darstellung des Verknüpfungsprozesses (reproduziert mit Erlaubnis von van Herk-Sukel et al. [29] auf unserer Website: http://record-statement.org/images/figure3.jpg).

**Abbildung 4:**
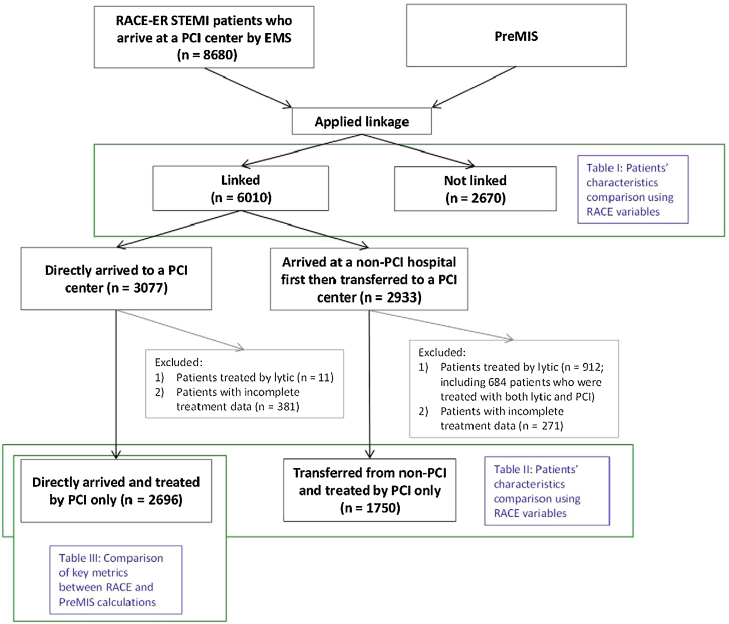
Verknüpfungsdiagramm kombiniert mit Teilnehmer-Flussdiagramm (reproduziert mit Erlaubnis von Fosbøl et al. [30] auf unserer Website: http://record-statement.org/images/figure4.jpg).

RECORD-PUNKT 6.3: Einige Möglichkeiten zur Veranschaulichung des Verknüpfungsprozesses werden beispielhaft in den Abbildung 2, Abbildung 3, Abbildung 4 gezeigt.

#### Erläuterung

RECORD-PUNKTE 6.1 und 6.2: Das Berichten der Validität der Identifizierungscodes/-algorithmen, die benutzt werden, um die Studienpopulation zu ermitteln, ist für eine transparente Berichterstattung von Beobachtungsstudien, die Routinedaten verwenden, unerlässlich. Zudem ermöglicht das Berichten von Codes/Algorithmen anderen Forschern, eine interne oder externe Validierung vorzunehmen.

Die verwendeten Methoden zur Identifizierung von Studienteilnehmern sollten klar und deutlich angegeben werden, einschließlich der Angabe, ob die Identifizierung auf einzelnen Codes, Algorithmen (Kombinationen von Datensätzen oder Codes), Verknüpfungen zwischen Datenbanken oder Freitext-Feldern basiert.

Das Risiko eines Bias durch falsche Klassifizierung („misclassification bias“) kann bei Studien mit Routinedaten, wie in vielen anderen epidemiologischen Studien, die Validität der Studienergebnisse gefährden [31]. Obgleich das Risiko falscher Klassifizierungen bei Studien, die Datenbanken mit großen Populationen verwenden, grösser ist, bieten diese Studien doch die Möglichkeit, seltene oder ungewöhnliche Krankheiten zu untersuchen [32]. Die Validierung der Identifizierungsmethoden wird zunehmend als unerlässlich angesehen für Studien, die Routinedaten verwenden. Dies gilt insbesondere für Krankheitscodes in Studien, die zu Abrechnungszwecken erhobene Verwaltungsdaten verwenden [33]. Externe Validierungsstudien beinhalten üblicherweise einen Vergleich der zur Identifizierung von Studienpopulationen verwendeten Codes oder Algorithmen mit einem Referenzstandard. Die gängigsten Referenzstandards sind Patientenakten, Befragungen von Patienten oder Ärzten sowie klinische Register [5], [34]. Zudem kann eine interne Validierung von Datenbanken durchgeführt werden, um sich überschneidende Datenquellen innerhalb einer einzigen Datenbank zu vergleichen [35]. Die Messgrößen für die Güte sind ähnlich zu denen, die in Studien für diagnostische Tests verwendet werden, und umfassen unter anderem Sensitivität, Spezifität, positive und negative prädiktive Werte oder Kappa-Koeffizienten [5], [34].

Wir empfehlen daher, bei Beobachtungsstudien, die Routinedaten verwenden, Details über die externe oder interne Validierung der Identifizierungscodes/-algorithmen in den Methodenteil des Manuskripts aufzunehmen. Wurden bereits zuvor eine oder mehrere Validierungsstudien durchgeführt, sollten diese referenziert werden. Wurden keine solchen Validierungsstudien durchgeführt, sollte dies ausdrücklich angegeben werden. Des Weiteren sollte die Güte der Identifizierungsmethoden (unter Verwendung gängiger Begriffe der diagnostischen Güte) und deren Funktionstüchtigkeit in der untersuchten Subpopulation kurz diskutiert werden. Wurden Validierungen im Rahmen der betreffenden Beobachtungsstudie durchgeführt, empfehlen wir den Autoren, die veröffentlichten Leitlinien für das Berichten von Validierungsstudien zu verwenden [5]. Es ist wichtig, anzugeben, ob die Validierung in einer anderen Quell- oder Datenbankpopulation als der für die aktuelle Studie ausgewählten erfolgte, da die Codes in verschiedenen Populationen oder Datenbanken unterschiedlich funktionieren können [36]. Falls darüber hinaus Probleme mit dem verwendeten Referenzstandard wie Unvollständigkeit oder Ungenauigkeit bekannt sind, sollten sie berichtet und überdies als Einschränkung diskutiert werden. Die Autoren sollten diskutieren, welche Auswirkungen die Verwendung ausgewählter Codes/Algorithmen beim Identifizieren von Studienpopulationen und –zielgrößen hat und welche Auswirkungen das Risiko falscher Klassifizierungen auf die Studienergebnisse haben könnte. Besonders wichtig ist es, zu diskutieren, welche Auswirkungen es hat, wenn in einer Validierungsstudie eine andere als die in der Studie untersuchten Studienpopulation verwendet wurde.

RECORD-PUNKT 6.3: Ein Flussdiagramm oder andere grafische Darstellung kann nützliche Informationen über den Verknüpfungsprozess vermitteln und eine womöglich langwierige Beschreibung vereinfachen. Solche Darstellungen können die wichtigsten Daten wie Informationen über den Anteil und die Merkmale der verknüpften und nicht verknüpften Einzelpersonen wiedergeben. Die Leser sollten in der Lage sein, den Anteil der erfolgreich verknüpften Datenbankpopulation sowie die Repräsentativität der sich ergebenden Studienpopulation zu ermitteln. Flussdiagramme zu Verknüpfungen können entweder als alleinstehende Diagramme (z. B. Venn- oder Flussdiagramme) oder in Kombination mit einem Teilnehmer-Flussdiagramm verwendet werden, wie es von STROBE empfohlen wird. Solche grafischen Darstellungen können viele verschiedene Formate benützen; wir empfehlen daher kein spezifisches.

### Methoden (Variablen)

RECORD-PUNKT 7.1: Eine vollständige Liste der zur Klassifizierung von Expositionen, Zielgrößen, Confoundern und Effektmodifikatoren verwendeten Codes und Algorithmen sollte wiedergegeben werden. Wenn dies nicht möglich ist, sollte erklärt werden warum.

### Beispiele

1. Hardelid et al. gaben alle Codes in der Tabelle S1 im Datensupplement 2 wieder [37].
2. Murray et al. gaben alle Codes für Risiko-Gruppen im Anhang S1 wieder [38].

#### Erläuterung

Wie bei den Codes/Algorithmen, die zur Identifizierung der Studienpopulation verwendet werden, machen die Codes/Algorithmen zur Klassifizierung von Expositionen, Zielgrößen, Confoundern oder Effektmodifikatoren die Forschung für möglichen Bias durch falsche Klassifizierung anfällig. Damit das Replizieren, Bewerten sowie Vergleichen mit anderen Studien möglich wird, empfehlen wir, dass im Manuskript, in einem Online-Anhang und/oder auf einer externen Website, eine Liste verfügbar gemacht wird, die alle diagnostischen, prozeduralen, medikamentenspezifischen oder sonstigen Codes enthält, die zur Durchführung der Studie verwendet wurden. Für Routinedaten aus Umfragen sollten die den Studienteilnehmern gestellten Fragen mit dem genauen Wortlaut bereitgestellt werden. Angesichts des Risikos von Bias durch falsche Klassifizierung bei jeder Forschung, einschließlich solcher, die Routinedaten verwendet [31], sollten die Autoren genug Informationen geben, um ihre Forschung reproduzierbar und das Bias-Risiko erkennbar zu machen. Validierungsstudien können im Artikel-Manuskript beschrieben oder eine Referenz zu anderem veröffentlichten oder Online-Material angegeben werden. Wie oben dargelegt, sollte von den Autoren angegeben werden, ob die Validierungsstudie mit einer anderen Quell- oder Datenbankpopulation als der in der vorliegenden Studie untersuchten durchgeführt wurde.

Uns ist bewusst, dass Forscher in einigen Fällen die in einer Publikation verwendeten Codelisten und Algorithmen nicht zur Verfügung stellen können, da es sich um durch Eigentums- oder Urheberrechte, Patente oder anderweitige Gesetze geschützte Informationen handelt. Beispielsweise wurden einige Indices für die Adjustierung von Komorbiditäten von gewinnorientierten Unternehmen entwickelt und an Forscher zur Verwendung im akademischen Forschungsumfeld verkauft [39], [40]. In diesen Fällen haben sich die Autoren bei der Erfassung, Bereinigung und/oder Verknüpfung von Daten möglicherweise auf Datenlieferanten oder vertrauenswürdige Dritte gestützt. Sie sollten dann jedoch ausführlich begründen, warum es nicht möglich ist, Codelisten oder andere Einzelheiten über die Vorgehensweise bei der Identifizierung von Personen oder Erkrankungen wiederzugeben, und sich bemühen, die Kontaktdaten der Gruppe beizufügen, die an den Listen Eigentumsrechte hat. Des Weiteren sollten die Autoren ansprechen, wie sich das Nichtbereitstellen dieser Informationen auf die Replikation und Evaluierung der Forschung durch den Leser auswirken kann. Im Idealfall sollten diese Dritten detaillierte Informationen über die Erhebung, Bereinigung oder Verknüpfung der Daten bereitstellen. Von einer verbesserten Kommunikation zwischen Datenlieferanten und -nutzern könnten beide Parteien profitieren.

Manche haben argumentiert, dass die Codelisten geistiges Eigentum der Forscher sind. Eine Veröffentlichung dieser Listen könnte es anderen Forschern ermöglichen, diese für ihre eigene Forschung zu verwenden und so die Autoren ihres geistigen Eigentums und der Anerkennung für die Erstellung der Codeliste zu berauben. Wir sind der Meinung, dass diese Auffassung nicht dem wissenschaftlichen Standard der Transparenz in Bezug auf die Replizierbarkeit von Forschung entspricht. Daher empfehlen wir, dass die vollständigen Codelisten, mit Ausnahme solcher, die gesetzlich oder vertraglich geschützt sind, veröffentlicht werden.

Wir sind uns bewusst, dass eine Veröffentlichung in einem Artikel in Papierformat aufgrund der Wörterzahl- und Platzbeschränkungen vieler Fachzeitschriften und der potenziellen Länge der Codelisten/Algorithmen nicht immer möglich ist. Stattdessen können Detailinformationen als Text, Tabellen oder als Anhang in Form eines Online-Supplements hinterlegt werden. Dies kann auf der Website der Fachzeitschrift, auf Servern, die von den Autoren oder anderen Personen auf Dauer betrieben werden oder im Datenbestand Dritter (z. B. Dryad oder Figshare) erfolgen. Die Text- und Referenzabschnitte des Manuskripts sollten ausführliche Informationen dazu enthalten, wie auf die Codelisten zugegriffen werden kann. Code-Repositorien wie ClinicalCodes.org erscheinen vielversprechend für die Dokumentation und Transparenz von Codes, die in der Forschung mit Gesundheitsdaten verwendet werden [41]. Werden die Codes in Online-Supplementen auf der Website der Fachzeitschrift oder auf einer externen Website, die von den Autoren bereitgestellt wird, veröffentlicht, sollte der Link im Hauptartikel der Fachzeitschrift erscheinen. Die Veröffentlichung auf der Website einer Fachzeitschrift oder auf PubMed Central (http://www.ncbi.nlm.nih.gov/pmc/) erhöht die Wahrscheinlichkeit, dass das Supplement verfügbar bleibt, solange die Fachzeitschrift existiert. Ist die Veröffentlichung auf einer externen privaten oder institutionellen Website die einzige Option, empfehlen wir, diese Listen nach Veröffentlichung des Zeitschriftenartikels mindestens zehn Jahre lang zur Verfügung zu stellen. Wurde die URL-Adresse geändert, ist eine automatische Weiterleitung von der alten Adresse der Website erforderlich. Dank dieser Maßnahmen können so auch zukünftige Leser des Artikels auf die vollständigen Codelisten zugreifen.

Neben den Codelisten, die im Artikel (oder einem Online-Anhang) zur Verfügung gestellt werden, sollten die Autoren auch erörtern, ob die Wahl der in der Studie verwendeten Codes/Algorithmen zu Bias führen kann. Dabei kann es sich unter anderem um Bias durch falsche Klassifizierung, „Ascertainment bias“ und Bias aufgrund fehlender Daten handeln. Wurden Sensitivitätsanalysen mit verschiedenen Code-/Algorithmus-Sets durchgeführt, sollten diese ebenfalls beschrieben und evaluiert werden. Die Diskussion über möglichen Bias kann auch im Zusammenhang mit anderen Bestandteilen der RECORD- und STROBE-Checklisten erfolgen, wie etwa der Auswahl der Studienteilnehmer und der (ggf. fehlenden) Validierung von Codes.

### Methoden (Statistische Methoden)

#### Datenzugriff und Bereinigungsmethoden

RECORD-PUNKT 12.1: Die Autoren sollten beschreiben, inwieweit die Forscher Zugang zur Datenbank hatten, die für die Zusammenstellung der Studienpopulation verwendet wurde.

RECORD-PUNKT 12.2: Die Autoren sollten Angaben zu den Methoden zur Datenbereinigung machen, die in der Studie verwendet wurden.

#### Verknüpfung

RECORD-PUNKT 12.3: Geben Sie an, ob im Rahmen der Studie eine Verknüpfung auf Personenebene, auf institutioneller Ebene oder andere Datenverknüpfungen zwischen zwei oder mehr Datenbanken durchgeführt wurden. Die Verknüpfungstechniken und Methoden zur Evaluierung der Verknüpfungsqualität sollten angegeben werden.

#### Beispiele

RECORD-PUNKT 12.1: Die folgenden Artikel beschreiben den Zugang zu einer Untergruppe der *UK General Practice Research Database* (GPRD).

1. „Für Projekte, die über die Lizenzvereinbarung des *Medical Research Council* finanziert werden, beschränkt die GPRD die Datensätze auf 100.000 Personen. Aufgrund dieser Beschränkung ist eher eine Fallkontrollstudie als eine Kohortenstudie erforderlich, um sicherzustellen, dass wir eine ausreichende Anzahl von Krebsfällen für jedes einzelne Symptom identifiziert haben…“ [42].
2. „Aus der *General Practice Research Database* wurde mit einer Lizenz des *Medical Research Council* für akademische Einrichtungen eine Zufallsstichprobe … gezogen [43].

RECORD-PUNKT 12.2: Beispiel für eine Beschreibung der Methoden zur Datenbereinigung [44]:

„Die Vollständigkeit der zur Verknüpfung verwendeten gemeinsamen Identifikatoren variierte zwischen den Datensätzen und über die Zeit (die Identifikatoren der letzten Jahre waren vollständiger). Bei LabBase2 variierten die Identifikatoren pro Einheit. Bei PICANet [Paediatric Intensive Care Audit Network] waren „Geburtsdatum“ und „Krankenhausnummer“ zu 100 % und die Mehrzahl der anderen Identifikatoren zu > 98 % vollständig, mit Ausnahme der Nummer des NHS (National Health Service) (zu 85 % vollständig). Für beide Datensätze wurde eine Datenbereinigung und -aufbereitung durchgeführt: NHS- oder Krankenhausnummern wie „Unbekannt“ oder „9999999999“ wurden auf Null gesetzt; generische Namen (z. B. „Baby“, „Twin 1“, „Infant Of“) wurden auf Null gesetzt; für mehrteilige Nachnamen und Vornamen wurden verschiedene Variablen erstellt; Postleitzahlen, die mit „ZZ“ beginnen (keine Postleitzahl in GB), wurden auf Null gesetzt.“

  

RECORD-PUNKT 12.3: Die folgenden Auszügen aus Artikeln sind Beispiele für gutes Berichten über die Ebenen der Datenverknüpfung, verwendete Verknüpfungstechniken und -methoden sowie die zur Evaluierung der Verknüpfungsqualität verwendeten Methoden:

1. „Wir haben Bescheinigungen über Lebend- und Totgeburten zu einer chronologischen Ereigniskette verknüpft, die - mit Ausnahme von Schwangerschaftsabbrüchen und ektopischen Schwangerschaften - die Fortpflanzungsvorgeschichte der einzelnen Frauen darstellte“ [45].
2. Zwei Artikel enthalten ausgezeichnete Beschreibungen von Verknüpfungen, die speziell für die Studie, über die berichtet wird, erstellt wurden [44], [45]. In dem Artikel von Harron et al. [44] wurde eine detaillierte Erläuterung zur Verknüpfung mit grafischer Darstellung des Matching-Prozesses gegeben. Zudem werden die Methoden zur Berechnung der Wahrscheinlichkeit der Verknüpfung beschrieben: „Die Match-Wahrscheinlichkeiten P (M | Übereinstimmungsmuster) wurden zur Schätzung der Wahrscheinlichkeit eines Matches bei Übereinstimmung eines gemeinsamen Satzes von Identifikatoren berechnet. Dadurch wurde die Annahme der Unabhängigkeit zwischen den Identifikatoren umgangen. Die Wahrscheinlichkeiten wurden abgeleitet, indem die Anzahl der Verknüpfungen durch die Gesamtanzahl der Paare für jedes Übereinstimmungsmuster (basierend auf im Trainingsdatensatz identifizierten wahrscheinlichen Verknüpfungen) geteilt wurde. Wenn beispielsweise bei 378 Vergleichspaaren eine Übereinstimmung hinsichtlich Geburtsdatum und Soundex besteht, jedoch keine Übereinstimmung hinsichtlich des Geschlechts vorliegt, und es sich bei 312 dieser Paare um wahrscheinliche Verknüpfungen handelt, läge die Match-Wahrscheinlichkeit für das Übereinstimmungsmuster [1,1,0] bei 312 : 378 = 0,825″ [44]. Der Artikel von Adams et al. enthält zudem eine detaillierte Erläuterung des Verknüpfungsprozesses: „Die deterministische Verknüpfung umfasste die Phase I mit sechs Verarbeitungsschritten, bei denen Verknüpfungsketten gebildet und einzelne (zuvor unverknüpfte) Datensätze den Ketten hinzugefügt wurden. Daraufhin folgte Phase n, die mehrere Durchläufe der Datei zur Kombination von Ketten mit derselben Mutter umfasste“ [45].
3. Sollte eine Studie hingegen auf zuvor verknüpfte Daten verweisen, ist es eventuell sinnvoll, eine frühere Publikation zu referenzieren: „Die Datensätze beider Datenbanken wurden mithilfe von Geburtsdatum, Geschlecht und Postleitzahl mit den kommunalen Melderegistern und anschließend miteinander verknüpft. Die Verknüpfung wurde von *Statistics Netherlands* durchgeführt und in früheren Publikationen beschrieben“ [20].
4. Nachfolgend ein Beispiel für gutes Berichten von Merkmalen von verknüpften und nicht verknüpften Personen: „Für die Zwecke dieser Publikation werden nicht gematchte Datensätze der ISC (Inpatient Statistics Collection) als ISC-Reste, nicht gematchte Datensätze der MDC (Midwives Data Collection) als MDC-Reste und verknüpfte Paare als gematchte Datensätze bezeichnet…. Ausgewählte Variablen, die in beiden Datensätzen vorhanden waren, wurden zwischen den drei Gruppen (ISC-Reste, MDC-Reste und gematchte Datensätze) verglichen“ [46].

#### Erläuterung

RECORD-PUNKTE 12.1 und 12.2: Wenn die Erstellung der Studienpopulation von Datenanalysten vorgenommen wird, die nicht mit den Besonderheiten des Aufbaus einer Kohorte oder den Studienzielen vertraut sind, kann es zu Fehlern kommen. Folglich sollte angegeben werden, in welchem Umfang die Autoren Zugriff auf die Datenbank hatten. Die Beschreibung der Methoden zur Datenbereinigung in den verschiedenen Studienabschnitten sollte auch Angaben zu den Methoden für das Suchen nach fehlerhaften oder fehlenden Daten einschließlich Wertebereichsprüfungen, Überprüfungen auf doppelt vorhandene Datensätze und Umgang mit wiederholten Messungen beinhalten [47], [48]. Weitere zu berichtende Methoden könnten die Untersuchung von Häufigkeitsverteilungen, Kreuztabellierungen von Daten und grafische Darstellungen oder den Einsatz von statistischen Methoden zur Erkennung von Ausreißern umfassen [49]. Zudem könnten weitere Informationen zur Fehlerdiagnose einschließlich Definitionen der Plausibilität und der Fehlerbehandlung bei der Analyse angegeben werden. Eine klare und transparente Beschreibung der Methoden zur Datenbereinigung ist wichtig, da die Wahl dieser Methoden die Studienergebnisse, die Wiederholbarkeit der Studie und auch die Nachvollziehbarkeit der Studienergebnisse beeinflussen könnte [50].

RECORD-PUNKT 12.3: Bei Verknüpfungsstudien schlagen wir vor, den geschätzten Anteil gelungener Verknüpfungen, die Verwendung deterministischer versus probabilistischer Verknüpfungen, die Qualität und den Typ der für die Verknüpfung verwendeten Variablen und die Ergebnisse aller Validierungen von Verknüpfungen anzugeben. Wurde speziell für die Studie eine Verknüpfung von Datensätzen zwischen Datenbanken durchgeführt, sollte über die Methoden der Verknüpfung und die Evaluierung der Qualität der Verknüpfung berichtet werden, und angegeben werden, wer die Verknüpfung vorgenommen hat. Falls vorhanden, sollten Informationen über Blocking-Variablen, die Vollständigkeit von Verknüpfungsvariablen, Regeln der Verknüpfung, Schwellenwerte und über manuelle Überprüfungen bereitgestellt werden [44]. Wurde die Verknüpfung vor der Studie (d. h. für frühere Studien oder zum allgemeinen Gebrauch) oder von einem externen Anbieter, z. B. von einem Datenverknüpfungszentrum, vorgenommen, so ist eine Referenz erforderlich, welche die Datenquelle und die Verknüpfungsmethoden beschreibt.

Damit der Leser die Auswirkungen jeglicher Verknüpfungsfehler und damit verbundenem Bias beurteilen kann, sind Angaben zu den Verknüpfungsmethoden und die Evaluierung ihres Erfolgs von entscheidender Bedeutung [51]. Insbesondere sollte der Leser erfahren, ob die verwendete Verknüpfungsart deterministisch und/oder probabilistisch war; so kann er beurteilen, ob die Verknüpfung durch fehlerhaftes oder fehlendes Matching beeinträchtigt wurde. Wenn ein eindeutiger Identifikator in den unterschiedlichen Datenquellen verfügbar ist, ist eine deterministische Verknüpfung sinnvoll. Ist ein solcher Identifikator nicht verfügbar, ist eine Beschreibung der angewandten Regeln der Datensatzverknüpfung (oder der statistische Verknüpfungsschlüssel) entscheidend. Im Gegensatz dazu werden bei der probabilistischen Verknüpfung mehrere Identifikatoren mitunter mit verschiedenen Gewichtungen verwendet und über einem bestimmten Schwellenwert wird eine Übereinstimmung angenommen. Es können auch gemischte Methoden zur Anwendung kommen. Zum Beispiel kann für manche Datensätze eine deterministische Verknüpfung verwendet werden, während beim Fehlen von eindeutigen Identifikatoren für andere Datensätze eine probabilistische Verknüpfung angewandt wird. Ein sog. Verknüpfungsbias („Linkage bias“) kann vorkommen, wenn die Wahrscheinlichkeit eines Verknüpfungsfehlers (z. B. falsche und fehlende Matches) mit Variablen von Interesse assoziiert sind. Beispielsweise können die Verknüpfungsraten je nach Patientenmerkmalen, z. B. Alter, Geschlecht und gesundheitlicher Zustand, variieren. Selbst geringe Fehler im Verknüpfungsprozess können zu Bias und Ergebnissen führen, die die zu untersuchenden Assoziationen über- oder unterschätzen [52]. Die Autoren sollten über Verknüpfungsfehler berichten und dabei Standardverfahren wie Vergleiche mit Gold-Standards oder Referenzdatenbeständen, Sensitivitätsanalysen und Vergleiche der Merkmale verknüpfter und nicht verknüpfter Daten verwenden [53]. Wenn über Verknüpfungsfehler berichtet wird, können Leser die Qualität der Verknüpfung und möglichen Bias aufgrund von Verknüpfungsfehlern bestimmen.

### Ergebnisse (Teilnehmer)

RECORD-PUNKT 13.1: Beschreiben Sie ausführlich die Auswahl der in die Studie aufgenommenen Personen (d. h. die Auswahl der Studienpopulation) einschließlich des Filterns aufgrund von Datenqualität, Datenverfügbarkeit und Verknüpfung. Die Auswahl der eingeschlossenen Personen kann im Fließtext und/oder mit Hilfe des Studienflussdiagramms beschrieben werden.

#### Beispiel

**Abbildung 5:**
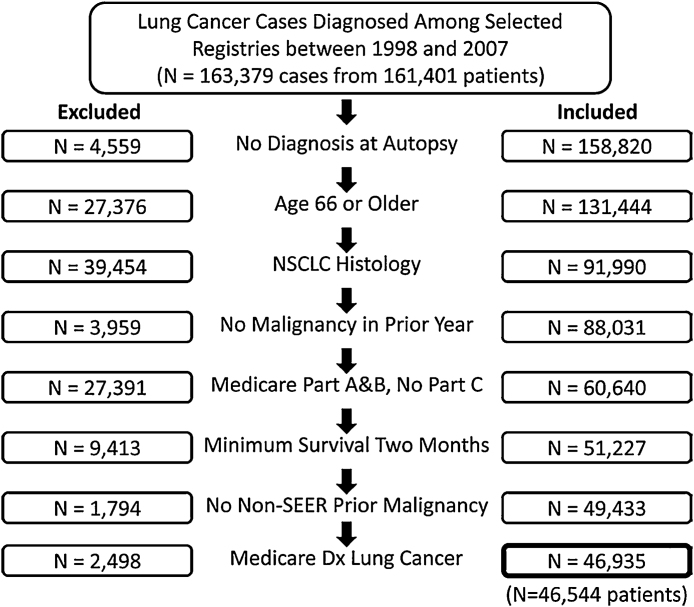
Beispiel Flussdiagramm Studie Ein- und Ausschlusskriterien mit Genehmigung von Dinan et al. [54] (Website: http://record-statement.org/images/figure5.jpg).

Der folgende Auszug enthält ein Beispiel für gutes Berichten:

„Wir identifizierten 161.401 Medicare-Versicherte, bei denen laut SEER-Registern (Surveillance, Epidemiology, and End Results) zwischen 1998 und 2007 in mindestens einem Fall ein Lungen- und Bronchialkarzinom diagnostiziert wurde. Unter diesen Patienten ermittelten wir 163.379 gesonderte Diagnosen von mehrfachen Lungenkarzinomen. (Einige Patienten hatten zwei primäre Lungenkarzinome, die im Verlauf der Studie in einem Zeitabstand von über einem Jahr auftraten). Abbildung 5 zeigt eine Abweichung der letzten Kohorte von 46.544 Patienten mit 46.935 nicht-kleinzelligen Lungenkarzinomen (Non-Small Cell Lung Cancer, NSCLC) [54].“

#### Erläuterung

Die Autoren sollten eine Abweichung der Studienpopulation(en) von den ursprünglichen Datenbanken mit den Routinedaten klar beschreiben. Unterschiede zwischen der Studienpopulation und der Datenbankpopulation müssen dokumentiert werden, damit etwaige Ergebnisse übertragen werden können (siehe auch RECORD-Punkt 6.1). Forscher, die Routinedatenquellen verwenden, beschränken häufig die Studienpopulation aufgrund von Faktoren wie der Qualität der verfügbaren Daten. Wenn beispielsweise die Studiendauer auf einen Zeitraum mit annehmbarer Datenqualität beschränkt wird, kann dies zu einem Ausschluss von möglichen Studienteilnehmern führen. Unter Umständen werden Arztpraxen mit inkonsistenter Eingabe in elektronische Patientenakten ausgeschlossen oder es wird gewartet, bis diese Praxen die Daten konsistent eingeben [38], [55]. Eine Studienpopulation kann auch basierend auf der Verfügbarkeit von Daten beschränkt werden. So werden beispielsweise in Studien, die Daten der US-amerikanischen Medicare verwenden, Versicherte, die aktuell bei einer Health Maintenance Organization registriert sind, häufig aufgrund fehlender Aufzeichnungen von klinischen Ereignissen ausgeschlossen [54], [56]. Bei der Verwendung von Datenquellen, in denen sich die Aufnahmekriterien („eligibility“) über die Zeit verändern kann (z. B. Versicherungsdatenbanken) müssen die Forscher eindeutige Angaben darüber machen, wie die Aufnahmekriterien definiert wurden und entsprechende Änderungen gehandhabt wurden. Wenn eine Studie verknüpfte Routinedaten verwendet, ist die Studienpopulation häufig kleiner, da sie auf Einzelpersonen, für die verknüpfte Daten verfügbar sind, beschränkt ist [57]. Zudem können aus methodischen Gründen stark eingeschränkte Kohorten verwendet werden, um Ursachen von Confounding auszuschalten.

Die für den Erhalt der endgültigen Studienpopulation(en) ergriffenen Massnahmen, Ein- und Ausschlusskriterien und der tatsächliche Ein- und Ausschluss von Studienteilnehmern in den verschiedenen Phasen der Kohortenerstellung und -analyse sollten im Fließtext des Manuskripts oder mit Hilfe eines geeigneten Flussdiagramms definiert werden. Studienpopulationen können unter Verwendung verschiedener Codes und/oder Algorithmen (siehe RECORD-Punkt 6.1) erstellt werden. Dabei können sich Unterschiede in der Verwendung von Codes im Verlauf der Zeit auf die Studienpopulation auswirken [58], [59]. Studien verwenden möglicherweise auch mehrere Falldefinitionen, die mehr oder weniger sensitiv/spezifisch sind, was sich auf nachfolgende Analysen auswirken kann. Eine Beschreibung dieser Schritte ist zur Bewertung der externen Validität von Studienergebnissen und, unter bestimmten Umständen, zur Bewertung eines möglichen Selektionsbias wichtig. Um die möglichen Auswirkungen eines Fehlens von Daten auf die Repräsentativität der Studienpopulation zu bewerten, können Sensitivitätsanalysen berichtet werden. Wenn Informationen über die Auswahl der Studienpopulation(en) aus der ursprünglichen Datenbank bereitgestellt werden, hilft dies auch, eine Studie zu replizieren. Möglicherweise wurden auch zusätzliche Analysen für verschiedene Studienpopulationen durchgeführt, die in Online-Anhängen berichtet werden können.

### Diskussion (Einschränkungen)

RECORD-PUNKT 19.1: Erörtern Sie, was es bedeutet, Daten zu verwenden, die nicht zur Beantwortung spezifischer Forschungsfragen erhoben bzw. gesammelt wurden. Diskutieren Sie dabei auch Bias durch falsche Klassifizierung, Confounding durch ungemessene Faktoren, fehlende Daten und sich mit der Zeit verändernder Aufnahmekriterien, soweit diese die Studie betreffen, über die berichtet wird.

### Beispiele

Die folgenden Artikel beschreiben Einschränkungen beim Verwenden von Verwaltungsdaten:

1. „Drittens handelte es sich bei dieser Studie um eine retrospektive, auf Rechnungsforderungen basierende Analyse. Es konnten nur Positronen-Emissions-Tomographie (PET) Aufnahmen, die von Medicare bezahlt wurden, in der Analyse ermittelt werden. Um den Anteil von versäumten Rechnungsforderungen zu minimieren, wurden alle Analysen auf Medicare-Versicherte beschränkt, die in den 12 Monaten vor und nach der Diagnose sowohl über Medicare Part A als auch Medicare Part B versichert waren und keinem Managed-Care-Programm angehörten bzw. über Medicare Part C versichert waren. Viertens ist es wahrscheinlicher, dass Patienten im SEER-Register nicht weißer Hautfarbe sind, in Gebieten mit weniger Armut oder in der Stadt leben, wodurch die Generalisierbarkeit der Ergebnisse eingeschränkt sein kann. Fünftens basierte das Krankheitsstadium während der Studiendauer auf SEER-Daten, die über einen Zeitraum von vier Monaten oder bis zum ersten operativen Eingriff erfasst wurden. Im Jahr 2004 wurde die Datenerhebung für SEER auf das gemeinschaftliche Staging-System geändert. Es ist unklar, inwieweit unsere Ergebnisse mit diesem neueren Ansatz abweichen würden“ [54].
2. „Trotz mehrerer Stärken der SEER-Medicare-Daten, wie eines vergleichbar großen Stichprobenumfangs, der Generalisierbarkeit auf die US-Population und den detaillierten Informationen über Verschreibungen, war unsere Studie aufgrund fehlender Labordaten zu Cholesterin, Triglyceriden und dem Blutzuckerspiegel eingeschränkt. Diese hätten Informationen über das Ausmaß von Stoffwechselstörungen in der Bevölkerung liefern können … Mit verfügbaren Labordaten hätte das residuale Confounding durch die Schwere der Stoffwechselstörung verringert werden können. Auch fehlten uns detailliertere Daten zur Progression der Krebserkrankung. Da Patienten mit einer kurzen Lebenserwartung eine Statin-Behandlung möglicherweise vorenthalten bzw. diese bei ihnen abgesetzt wurde, könnte dies ein Confounder in der Assoziation zwischen Gabe von Statinen und Todesfällen gewesen sein“ [60].

### Erläuterung

Routinedaten werden in der Regel nicht aufgrund einer speziellen, a priori formulierten Forschungsfrage gesammelt. Die Gründe für die Datenerfassung können unterschiedlicher Natur sein. Die Schlussfolgerungen der Forscher sind durch zahlreiche Arten von Bias gefährdet. Dazu zählen die üblichen Bias-Quellen im Zusammenhang mit Beobachtungsstudien, aber auch einige, die spezifischer für Beobachtungsstudien sind, die Routinedaten verwenden. Folgende Punkte sollten von den Autoren als mögliche Bias-Quellen erörtert werden: (1) Codes oder Algorithmen zur Ermittlung von Studienpopulationen, Zielgrößen, Confounder oder Effektmodifikatoren (Bias durch falsche Klassifizierung), (2) fehlende Variablen (ungemessene Confounder); (3) fehlende Daten und (4) Änderungen hinsichtlich der Aufnahmekriterien im Verlauf der Zeit.

Der ursprüngliche Grund für die Erhebung von Routinedaten kann sich auf die Qualität der Daten und ihre Eignung für die untersuchten Forschungsfragen auswirken. So können zum Beispiel Register, die für retrospektive Analysen verwendet werden, über eine bessere Qualitätskontrolle verfügen als Organisationen, die andere Typen von Routinedaten sammeln, obgleich dies auch anders sein kann. In ähnlicher Weise werden einige Verwaltungsdaten einer gründlichen und andere Daten gar keiner Qualitätskontrolle unterzogen. Verwaltungsdaten sind beim sog. „Upcoding“ oder opportunistischen Coding besonders fehleranfällig. Basiert die Krankenhausvergütung beispielsweise auf der Komplexität der Erkrankungen im Patientenkollektiv, maximieren die Krankenhäuser unter Umständen die Erstattung, indem sie den Patientenakten in großzügiger Weise mehr Codes für komplexe Krankheitsbilder hinzufügen [61]. Des Weiteren können sich Änderungen bei den Coding-Strategien auf die Validität oder Konsistenz der Daten auswirken. Beispielsweise kann die Einführung von Codes als Anreiz für die Verwendung von Abrechnungsdienstleistern die Wahrscheinlichkeit der Verwendung eines Codes über die Zeit ändern [62], [63]. Andere Codes werden möglicherweise gemieden, da sie zur Stigmatisierung von Patienten oder Sanktionen für die Anbieter führen [64]. Zudem können geänderte Versionen von Code-Klassifizierungssystemen (z. B. von der Internationalen Klassifikation der Krankheiten (ICD)-9 auf ICD-10) die Validität von Erhebungen unter Verwendung codierter Daten verändern [65], [66]. Abweichungen in der klinischen Praxis zwischen Krankenhäusern und Bevölkerungsgruppen können dazu führen, dass an bestimmten Orten und/oder Arztpraxen Laboruntersuchungen durchgeführt werden, was Auswirkungen auf einen Diagnose-Algorithmus zur Folge haben kann. Sollten irgendwelche dieser potenzielle Quellen von Bias durch falsche Klassifikation vorhanden sein, sollten diese als Einschränkungen der Studie diskutiert werden.

Residuales Confounding ist definiert als Confounding in Verbindung mit Variablen, die nicht in den zu untersuchenden Daten eingeschlossen sind, was Bias zur Folge haben kann [67]. Dies ist eine potenzielle Bias-Quelle in allen Beobachtungsstudien, jedoch insbesondere in Studien, die Routinedaten verwenden. Um sie zu analysieren, sind unter Umständen Variablen erforderlich, die zum Zeitpunkt der Planung der Datenbanken oder der Erfassung der Daten nicht in Betracht gezogen wurden. Es wurden verschiedene Methoden zur Berücksichtigung dieser potenziellen Bias-Quelle vorgeschlagen einschließlich Propensity-Scores [68], [69], [70], [71]. Jedoch können Propensity-Scores, wie auch die gewöhnliche Regressionsanalyse oder das standardmäßige Matching, nur ein Gleichgewicht zwischen den Studienteilnehmern hinsichtlich der in den Daten verfügbaren Variablen garantieren. Ein besonderer Typ ungemessenen Confoundings ist indikationsbedingtes Confounding („confounding by indication“). Dies stellt häufig ein Problem dar, wenn die Wirkung und Sicherheit (medikamentöser) Behandlungen mit Hilfe von Routinedaten untersucht werden. So kann die Prognose für Patienten, die eine (medikamentöse) Behandlung erhalten, besser oder schlechter ausfallen als die Prognose für Patienten, die keine (medikamentöse) Behandlung erhalten. Angaben zur Prognose und/oder Schwere der zugrundeliegenden Erkrankung stehen jedoch womöglich nicht im Datensatz zur Verfügung [72]. Probleme dieser Art sollten von den Autoren erörtert werden und die verwendeten Methoden zur Berücksichtigung dieser Probleme sollten (sofern möglich) berichtet werden.

Fehlende Daten können bei allen Beobachtungsstudien Schwierigkeiten bereiten; sie werden in der Box 6 des erläuternden STROBE-Artikels behandelt [10]. Fehlende Daten sind bei Routinedaten besonders problematisch, da die Datenerhebung von den Forschern nicht kontrolliert werden kann [73]. Fehlende Daten können zu Selektionsbias führen, wenn es fehlende Werte gibt bei Variablen, die zur Definition der Studienkohorte verwendet werden, oder fehlende Identifikatoren, welche die Verknüpfung von Datensätzen verhindern, besonders wenn die fehlenden Daten nicht zufällig verteilt sind. Fehlende Variablen stellen ähnliche Herausforderungen dar. Die Autoren sollten die fehlenden Variablen, von denen angenommen wird, dass sie ungemessenes Confounding erzeugen, den Grund für das Fehlen dieser Variablen, die möglichen Auswirkungen auf die Studienergebnisse sowie die zur Adjustierung für fehlende Variablen verwendeten Methoden präzise schildern. Beispielsweise hat der Raucherstatus eine großen Auswirkung auf die Schwere von Morbus Crohn und wurde mit den Ergebnissen der Behandlung dieser Krankheit in Zusammenhang gebracht. Allerdings ist der Raucherstatus selten in administrativen Gesundheitsdaten enthalten. Bei einer Studie, die die Assoziation zwischen sozioökonomischem Status und den Behandlungsergebnissen bei Morbus Crohn untersuchte, wurde der Raucherstatus als potenzieller ungemessener Confounder diskutiert [74]. Häufig werden fehlende Daten/Variablen erst nach Beginn der Studie mit Routinedaten entdeckt. Dies macht es erforderlich, dass die Forscher von ihrem ursprünglichen Studienprotokoll abweichen. Es sollten stets die Einzelheiten der Abweichungen vom Studienprotokoll, unabhängig vom Grund für die Abweichung, berichtet werden. Es sollten die Gründe für die Abweichungen sowie die Auswirkungen auf die Studie und die Schlussfolgerungen diskutiert werden.

Eine weitere mögliche Einschränkung sind Änderungen der Coding-Verfahren oder Aufnahmekriterien, die sich aufgrund einer Änderung der Zusammensetzung der Datenbankpopulation, Studienpopulation oder beiden über die Zeit ergeben. Die Definition der Datenbankpopulation kann sich aufgrund einer Vielzahl von Umständen ändern, z. B. wenn Arztpraxen, welche Patienten rekrutieren, die Arbeit mit der Datenbank beenden, andere Computer-Software verwendet wird oder die Kriterien zur Aufnahme in die Datenbank, z. B. in ein Register, geändert werden. Die Studienpopulation bei Verwendung administrativer Datenquellen (z. B. Versicherungsdatenbanken) kann sich verändern, wenn die Eignung von Personen aufgrund eines geänderten Arbeitsverhältnisses, Aufenthaltsstatus oder Gesundheitsdienstleisters nicht konstant ist. Auch eine veränderte Vorgehensweise bei der Codierung von Datensätzen (z. B. Upcoding oder Änderungen im Codierungssystem wie oben beschrieben) kann zu einer Veränderung der Studienpopulation führen [63], [75], [76]. Damit der Leser das Verzerrungspotenzial beurteilen kann, sollten die Autoren bei der Erörterung von Beschränkungen erläutern, wie eine Änderung der Aufnahmekriterien in der Analyse gehandhabt wurde. Wie in STROBE erläutert, sollte die Diskussion die Richtung und das Ausmaß möglichen Biases und die ergriffenen Maßnahmen zu seiner Handhabung umfassen.

### Weitere Informationen

RECORD-PUNKT 22.1: Die Autoren sollten darüber informieren, wie auf ergänzende Informationen wie Studienprotokoll, Rohdaten oder Programmcode zugegriffen werden kann.

### Beispiele

1. Der Artikel von Taljaard et al. enthält das vollständige Studienprotokoll für eine Studie, die den Canadian Community Health Survey benutzt hat [77].
2. In ihrem Artikel laden Guttmann et al. dazu ein, das Studienprotokoll anzufordern: „Datenaustausch: Der technische Anhang, der Plan bzw. das Protokoll zur Erstellung des Datensatzes und der statistische Code sind vom korrespondierenden Autor über E-Mail [E-Mail-Adresse] erhältlich“ [78].

#### Erläuterung

Wir unterstützen nachdrücklich die Verbreitung detaillierter Informationen über Studienmethoden und -ergebnisse. Sofern möglich, unterstützen wir die vor- oder gleichzeitige Veröffentlichung des Studienprotokolls, der Ergebnisse der Rohdaten und ggf. des Programmcodes. Diese Informationen helfen Peer-Reviewern und Lesern, die Validität der Studienergebnissen zu beurteilen. Den Forschern stehen eine Reihe von Möglichkeiten zur freien Veröffentlichung (Open Publication) solcher Daten zur Verfügung. Dazu gehört das ergänzende online verfügbare Material von Zeitschriften, persönliche und institutionelle Websites, wissenschaftliche Social Media-Websites (z. B. ResearchGate.net und Academia.edu), Datenrepositorien (z. B. Dryad oder Figshare) oder Open Data-Websiten von öffentlichen Stellen [79]. Uns ist bewusst, dass einige Forschungsorganisationen, Körperschaften, Einrichtungen oder Gesetze die freie Verfügbarkeit von Informationen dieser Art unter Umständen verbieten oder einschränken. Obwohl die Diskussion über Eigentumsrechte und die Verwendung dieses geistigen Eigentums außerhalb des Anwendungsbereichs der RECORD-Leitlinien liegt, sollte die Veröffentlichung solcher Daten grundsätzlich innerhalb der rechtlichen und ethischen Richtlinien der Institution der Forscher und unter der Anleitung der Redakteure der Fachzeitschriften erfolgen. Diese Informationen wären auch für andere Forscher hilfreich, die eventuell auf diese Daten zugreifen möchten, um die in dem Manuskript beschriebene Forschung zu replizieren, zu reproduzieren oder zu ergänzen. Ungeachtet des Formats oder Umfangs der verfügbaren ergänzenden Informationen empfehlen wir, im Manuskript eindeutige Angaben zum Speicherort dieser Informationen zu machen.

## Diskussion

Die RECORD-Leitlinien gelten speziell für Beobachtungsstudien, die Routinedaten verwenden. Sie sollen die STROBE-Leitlinien ergänzen und nicht ersetzen. RECORD wurde entwickelt als Leitfaden für Autoren, Redakteure von Fachzeitschriften, Peer-Reviewer und andere Interessengruppen, um die Transparenz und Vollständigkeit des Berichtens von Studien, die Routinedaten verwenden, zu fördern. Die Checkliste ist zur Verwendung durch alle Forscher gedacht, die solche Daten nutzen, und wir unterstützen die breit angelegte Verbreitung an alle interessierten Parteien. Wir erwarten, dass das Berichten von Studien, die Routinedaten verwenden, transparenter werden wird, wenn RECORD von den Fachzeitschriften angenommen und umgesetzt wird.

### Einschränkungen

Sowohl STROBE als auch RECORD sind ausschließlich für die Anwendung auf Beobachtungsstudien ausgelegt. Routinedaten werden jedoch gelegentlich für Forschung mit anderen Studiendesigns verwendet, wie beispielsweise für cluster-randomisierte Studien in der Versorgungsforschung. Außerdem kann es sinnvoll sein, Daten aus randomisierten Studien mit Verwaltungsdaten zu verknüpfen, um Teilnehmer für bestimmte Zielgrössen langfristig nachzubeobachten. Damit verbundene Studien würden nicht als beobachtend erachtet werden. So wie sich das Forschungsfeld weiterentwickelt, werden auch wir voraussichtlich, mit ähnlicher Methodik, RECORD auf andere Forschungsdesigns ausweiten.

Mit RECORD versuchen wir, die Interessen und Prioritäten der Stakeholder bestmöglich wiederzugeben. Dabei erkennen wir, dass sich die Methoden für die Durchführung von Forschungsarbeiten mit Routinedaten schnell ändern und eine zunehmende Zahl verschiedener Datentypen für solche Forschung verfügbar wird. So gibt es beispielsweise Mobile Gesundheitsapplikationen (mHealth-Apps) für immer mehr Smartphones und tragbare Technologien. Obwohl diese Datenquellen derzeit nur begrenzt zur Forschung benutzt werden, erwarten wir, dass die Verwendung dieser Daten in naher Zukunft rasch zunehmen wird und dass neue Methoden zum Umgang mit dieser Ressource entwickelt werden. Außerdem hat sich das Working Committee auf Gesundheitsdaten konzentriert, was andere für die Gesundheitsforschung verwendete Datenquellen (z. B. Umweltdaten, Finanzdaten etc.) ausschliesst. Daher enthält die RECORD-Checkliste möglicherweise nicht alle Themen, die in der Zukunft wichtig werden könnten. Zu gegebener Zeit könnte dies eine Überarbeitung erforderlich machen.

Wir unternahmen erhebliche Anstrengungen, um möglichst viele Interessengruppen in die Erstellung dieser Leitlinien einzubeziehen. So haben wir mittels öffentlicher Aufrufe und gezielter Einladungen versucht, verschiedene Stakeholder für eine Mitarbeit an diesen Leitlinien zu gewinnen [16]. Allerdings repräsentieren die meisten Stakeholder Regionen, die Routinedaten für Forschungszwecke verwenden; Vertreter aus Entwicklungsländern und nicht englischsprachigen Ländern sind unterrepräsentiert. Dennoch sind wir der Meinung, dass die Stakeholder-Gruppe repräsentativ für die derzeitigen Forscher und Nutzer der so erworbenen Kenntnisse war. Wir erhielten sehr viele Beiträge in den Umfragen und Rückmeldungen der Stakeholder. Die einzelnen Empfehlungen in den Leitlinien wurden aus Gründen der Machbarkeit von einem kleineren, aus 19 Mitgliedern bestehenden Working Committee formuliert, die sich dazu persönlich getroffen hat, wie in der Literatur vorgeschlagen [17]. Zukünftig werden technische Neuerungen und soziale Medien eine aktivere Teilnahme größerer Gruppen an den Treffen des Working Committees möglich machen.

### Zukünftige Ausrichtung und Engagement in der Community

Routinedaten aus dem Gesundheitswesen werden zunehmend verfügbar. Daher gehen wir davon aus, dass mehr und mehr auch Forscher aus Regionen, in denen auf solche Art von Daten derzeit noch nicht zugegriffen werden kann, eingebunden werden können. Wir hoffen, dass Interessierte via Webseite record-statement.org und Forum fortlaufend mit Kommentaren und Diskussionspunkten zu RECORD beitragen. Dies kann uns in der Zukunft bei einer formalen Überarbeitungen helfen. Mithilfe dieser Online-Community wird RECORD zu einem dynamischen Dokument, das an die Veränderungen in diesem Forschungsfeld angepasst werden kann.

Die Veröffentlichung einer Leitlinie für die Berichterstattung und die Anerkennung durch Fachzeitschriften allein sind nicht ausreichend, um das Berichten von Forschung zu verbessern [80]. Für einen messbaren Erfolg sind die Art und Weise, wie die Leitlinien von Forschern, Fachzeitschriften und Peer-Reviewern umgesetzt werden, von entscheidender Bedeutung [81]. Deshalb wird es im Online-Auftritt ein Diskussionsforum zur Umsetzung der Leitlinien geben. Wir würden es befürworten, wenn die Auswirkungen von RECORD auf das Berichten „im Feld“ beurteilt würde. So könnte sichergestellt werden, dass die Leitlinien einen messbaren Nutzen liefern.

## Schlussfolgerungen

Das RECORD-Statement ergänzt die STROBE-Kriterien für Beobachtungsstudien, die Routinedaten aus dem Gesundheitswesen verwenden. Mit Unterstützung der in Forschung und Publikationswesen Aktiven haben wir Leitlinien in Form einer Checkliste und dieses zugehörige erläuternde Dokument entwickelt. Es konnte gezeigt werden, das solche Leitlinien das Berichten von Forschungsergebnissen verbessert, was wiederum den Nutzern der Forschung ermöglicht, die Stärken, Einschränkungen und die Güte der Schlussfolgerungen einschätzen zu können [12], [82], [83], [84]. Wir gehen davon aus, dass sich RECORD mit der Weiterentwicklung neuer Forschungsmethoden im Feld verändern wird. Gleichwohl hoffen wir, dass es diese Leitlinien in den kommenden Jahren leichter machen werden, Forschung angemessen zu berichten. Wenn Autoren, Redakteure von Fachzeitschriften und Peer-Reviewer RECORD umsetzen, gehen wir davon aus, dass dies zu Transparenz, Reproduzierbarkeit und Vollständigkeit beim Berichten von Forschung mit Routinedaten aus dem Gesundheitswesen führt.

## Danksagungen

Diese Arbeit wurde von der Ethikkommission des *Children's Hospital of Eastern Ontario* genehmigt. Die Autoren danken den Stakeholdern, die an den Umfragen zur Priorisierung von Themen zur Aufnahme in die Checkliste beigetragen haben. Auch danken wir den Mitgliedern der STROBE-Initiative für ihre Anleitung und Unterstützung bei der Entwicklung von RECORD. Die Autoren danken den RECORD-Forschungskoordinatoren Pauline Quach und Danielle Birman herzlich für ihre Beiträge, sowie Andrew Perlmutar, dem Web-Designer und Administrator von record-statement.org. Die Autoren danken auch allen Stakeholdern für ihre Beiträge zur Erstellung dieser Leitlinien.

Mitglieder des RECORD Working Committee: Douglas Altman (Centre for Statistics in Medicine, Oxford University), Nicholas de Klerk (University of Western Australia), Lars G. Hemkens (University Hospital Basel), David Henry (University of Toronto and Institute for Clinical Evaluative Sciences, Toronto), Jean-Marie Januel (University of Lausanne), Marie-Annick Le Pogam (Institute of Social and Preventive Medicine, University Hospital of Lausanne), Douglas Manuel (Ottawa Hospital Research Institute, University of Ottawa), Kirsten Patrick (Redakteurin, *Canadian Medical Association Journal* [CMAJ]), Pablo Perel (London School of Hygiene and Tropical Medicine), Patrick S. Romano (University of California, Davis, Co-Chefredakteur, Health Services Research), Peter Tugwell (University of Ottawa, Chefredakteur, *Journal of Clinical Epidemiology*), Joan Warren (National Institutes of Health/National Cancer Institute), Wim Weber (Redakteur der europäischen Ausgabe von *BMJ*) und Margaret Winker (ehemals leitende Redakteurin Forschung, *PLOS Medicine*; derzeitige Schriftführerin, World Association of Medical Editors).

Übersetzung: Elsevier, Lars G. Hemkens (University Hospital Basel), Erik von Elm (Cochrane Schweiz, Universitätsspital Lausanne). Der ursprüngliche Artikel wurde in englischer Sprache veröffentlicht [85] und ist unter http://journals.plos.org/plosmedicine/article?id=10.1371/journal.pmed.1001885 verfügbar.

## Interessenkonflikt

Die Autoren erklären, dass kein Interessenkonflikt besteht.

## Figures and Tables

**Tabelle 1:** Das RECORD-Statement: Checkliste von Punkten in Erweiterung des STROBE-Statements, die in Beobachtungsstudien mit Routinedaten berichtet werden sollten.

	Nr.	STROBE-Punkte	RECORD-Punkte
**Titel und Abstract**			
	1	(a) Machen Sie das Studiendesign im Titel oder Abstract kenntlich, indem Sie dafür einen allgemein gebräuchlichen Begriff verwenden (b) Verfassen Sie für das Abstract eine aussagefähige und ausgewogene Zusammenfassung dessen, was in der Studie gemacht wurde und was herausgefunden wurde	RECORD 1.1: Der verwendete Datentyp sollte im Titel oder Abstract angegeben werden. Die Namen der verwendeten Datenbanken sollten, sofern möglich, aufgeführt werden. RECORD 1.2: Gegebenenfalls sollte die geografische Region und der Zeitrahmen, in dem die Studie durchgeführt wurde, im Titel oder Abstract angegeben werden. RECORD 1.3: Wurde für die Studie eine Verknüpfung von Datenbanken vorgenommen, sollte dies ausdrücklich im Titel oder Abstract angegeben werden.
**Einleitung**			
Hintergrund /Rationale	2	Erläutern Sie den wissenschaftlichen Hintergrund und die Rationale für die vorgestellte Studie	
Zielsetzungen	3	Geben Sie alle spezifischen Zielsetzungen einschließlich der (vorab festgelegten) Hypothesen an	
**Methoden**			
Studiendesign	4	Beschreiben Sie die wichtigsten Elemente des Studiendesigns möglichst früh im Artikel	
Rahmen	5	Beschreiben Sie den Rahmen (Setting) und Ort der Studie und machen Sie relevante zeitliche Angaben, einschließlich der Zeiträume der Rekrutierung, der Exposition, der Nachbeobachtung und der Datensammlung	
Studienteilnehmer	6	***(a)** Kohortenstudie*: Geben Sie die Einschlusskriterien, die Herkunft der Teilnehmer sowie die Methoden ihrer Auswahl an; beschreiben Sie die Methoden der Nachbeobachtung *Fallkontrollstudie*: Geben Sie die Einschlusskriterien und die Herkunft der Fälle und Kontrollen an sowie die Methoden, mit denen die Fälle erhoben und die Kontrollen ausgewählt wurden. Geben Sie eine Begründung (Rationale) für die Auswahl der Fälle und Kontrollen *Querschnittsstudie*: Geben Sie die Einschlusskriterien, die Herkunft der Teilnehmer sowie die Methoden ihrer Auswahl an ***(b)** Kohortenstudie*: Geben Sie für Studien, die Matching (Paarbildung) verwenden, die Matchingkriterien und die Anzahl der exponierten und der nicht exponierten Teilnehmer an *Fallkontrollstudie*: Geben Sie für Studien, die Matching (Paarbildung) verwenden, die Matchingkriterien und die Anzahl der Kontrollen pro Fall an	RECORD 6.1: Die Methoden für die Auswahl der Studienpopulation (wie verwendete Codes oder Algorithmen zur Identifizierung von Teilnehmern) sollten detailliert aufgelistet werden. Ist dies nicht möglich, sollte dies erklärt werden. RECORD 6.2: Für alle Studien zur Validierung der Codes und Algorithmen, die für die Auswahl von Teilnehmern verwendet wurden, sollten Referenzen angegeben werden. Wurde eine Validierung für diese Studie durchgeführt und nicht anderweitig veröffentlicht, sollten die Methoden und Ergebnisse detailliert dargestellt werden. RECORD 6.3: Wurden in der Studie Datenbanken verknüpft, sollte zur Darstellung des Verknüpfungsprozesses die Verwendung eines Flussdiagramms oder einer anderen grafischen Darstellung in Betracht gezogen werden. Dies sollte auch die Anzahl von Personen mit verknüpften Daten in den einzelnen Abschnitten enthalten.
Variablen	7	Definieren Sie eindeutig alle Zielgrößen, Expositionen, Prädiktoren, mögliche Confounder und Effektmodifikatoren; geben Sie gegebenenfalls Diagnosekriterien an	RECORD 7.1: Eine vollständige Liste der zur Klassifizierung von Expositionen, Zielgrößen, Confoundern und Effektmodifikatoren verwendeten Codes und Algorithmen sollte wiedergegeben werden. Wenn dies nicht möglich ist, sollte erklärt werden warum.
Datenquellen/Messmethoden	8	Geben Sie für jede in der Studie wichtige Variable die Datenquellen an und erläutern Sie die verwendeten Bewertungs- bzw. Messmethoden. Beschreiben Sie die Vergleichbarkeit der Messmethoden, wenn es mehr als eine Gruppe gibt	
Bias	9	Beschreiben Sie, was unternommen wurde, um möglichen Ursachen von Bias zu begegnen	
Studiengröße	10	Erklären Sie,wie die Studiengröße ermittelt wurde.	
Quantitative Variablen	11	Erklären Sie, wie in den Auswertungen mit quantitativen Variablen umgegangen wurde Wenn nötig, beschreiben Sie, wie Kategorien (Gruppierungen) gebildet wurden und warum	
Statistische Methoden	12	(a) Beschreiben Sie alle statistischen Methoden, einschließlich der Methoden, die für die Kontrolle von Confounding verwendet wurden (b) Beschreiben Sie Verfahren, mit denen Subgruppen und Interaktionen untersucht wurden (c) Erklären Sie, wie mit fehlenden Daten umgegangen wurde (d) *Kohortenstudie* – Erklären Sie gegebenenfalls, wie mit dem Problem des vorzeitigen Ausscheidens aus der Studie („loss to follow-up“) umgegangen wurde *Fallkontrollstudie* – Beschreiben Sie gegebenenfalls, wie das Matching (Paarbildung) von Fällen und Kontrollen bei der Auswertung berücksichtigt wurde *Querschnittsstudie* – Beschreiben Sie gegebenenfalls die Auswertungsmethoden, die die gewählte Strategie zur Stichprobenauswahl (Sampling strategy) berücksichtigen (e) Beschreiben Sie vorgenommene Sensitivitätsanalysen	
Datenzugriff und Reinigungs-methoden		entfällt	RECORD 12.1: Die Autoren sollten beschreiben, inwieweit die Forscher Zugang zur Datenbank hatten, die für die Zusammenstellung der Studienpopulation verwendet wurde. RECORD 12.2: Die Autoren sollten Angaben zu den Methoden zur Datenbereinigung machen, die in der Studie verwendet wurden.
Verknüpfung		entfällt	RECORD 12.3: Geben Sie an, ob im Rahmen der Studie eine Verknüpfung auf Personenebene, auf institutioneller Ebene oder andere Datenverknüpfungen zwischen zwei oder mehr Datenbanken durchgeführt wurden. Die Verknüpfungstechniken und Methoden zur Evaluierung der Verknüpfungsqualität sollten angegeben werden.
**Ergebnisse**			
Teilnehmer	13	(a) Geben Sie die Anzahl der Teilnehmer während jeder Studienphase an, z. B. die Anzahl der Teilnehmer, die potenziell geeignet waren, die auf Eignung untersucht wurden, die als geeignet bestätigt wurden, die tatsächlich an der Studie teilgenommen haben, deren Nachbeobachtung abgeschlossen wurde und deren Daten ausgewertet wurden (b) Geben Sie die Gründe für die Nicht-Teilnahme in jeder Studienphase an (c) Erwägen Sie die Darstellung in einem Flussdiagramm	RECORD 13.1: Beschreiben Sie ausführlich die Auswahl der in die Studie aufgenommenen Personen (d. h. die Auswahl der Studienpopulation) einschließlich des Filterns aufgrund von Datenqualität, Datenverfügbarkeit und Verknüpfung. Die Auswahl der eingeschlossenen Personen kann im Fließtext und/oder mit Hilfe des Studienflussdiagramms beschrieben werden.
Deskriptive Daten	14	(a) Beschreiben Sie Charakteristika der Studienteilnehmer (z. B. demographische, klinische und soziale Merkmale) sowie Expositionen und mögliche Confounder (b) Geben Sie für jede Variable die Anzahl der Teilnehmer mit fehlenden Daten an (c) *Kohortenstudie*: Fassen Sie die Nachbeobachtungszeit zusammen (z. B. Mittelwert und Gesamtzeitraum)	
Ergebnisdaten („outcome data“)	15	*Kohortenstudie:* Berichten Sie über die Anzahl der Zielereignisse oder statistischen Maßzahlen (z. B. Mittelwert und Standardabweichung) im zeitlichen Verlauf *Fallkontrollstudie*: Berichten Sie über Teilnehmerzahlen in jeder Expositionskategorie oder über statistische Maßzahlen der Exposition (z. B. Mittelwert und Standardabweichung) *Querschnittstudie:* Berichten Sie über die Anzahl der Zielereignisse oder statistische Maßzahlen (z. B. Mittelwert und Standardabweichung)	
Hauptergebnisse	16	(a) Geben Sie die unadjustierten Schätzwerte an und gegebenenfalls auch die Schätzwerte in denen Adjustierungen für die Confounder vorgenommen wurden sowie deren Präzision (z. B. 95%-Konfidenz-intervall); machen Sie deutlich, für welche Confounder adjustiert wurde und warum diese berücksichtigt wurden (b) Wenn stetige Variablen kategorisiert wurden, geben Sie die oberen und unteren Grenzwerte der einzelnen Kategorien an (c) Wenn relevant, erwägen Sie, für aussagekräftige Zeiträume Schätzwerte relativer Risiken auch als absolute Risiken auszudrücken	
Weitere Auswertungen	17	Berichten Sie über weitere vorgenommene Auswertungen, z. B. die Analyse von Subgruppen und Wechselwirkungen (Interaktionen) sowie Sensitivitätsanalysen	
**Diskussion**			
Hauptergebnisse	18	Fassen Sie die wichtigsten Ergebnisse in Hinsicht auf die Studienziele zusammen.	
Einschränkungen	19	Diskutieren Sie die Einschränkungen der Studie und berücksichtigen Sie dabei die Gründe für möglichen Bias oder Impräzision. Diskutieren Sie die Richtung sowie das Ausmaß jedes möglichen Bias	RECORD 19.1: Erörtern Sie, was es bedeutet, Daten zu verwenden, die nicht zur Beantwortung spezifischer Forschungsfragen erhoben bzw. gesammelt wurden. Diskutieren Sie dabei auch Bias durch falsche Klassifizierung, Confounding durch ungemessene Faktoren, fehlende Daten und sich mit der Zeit verändernder Aufnahmekriterien, soweit diese die Studie betreffen, über die berichtet wird.
Interpretation	20	Nehmen Sie eine vorsichtige übergreifende Interpretation der Resultate vor und berücksichtigen Sie dabei die Ziele und Einschränkungen der Studie, die Multiplizität der Analysen, die Ergebnisse anderer Studien und andere relevante Evidenz	
Generalisier-barkeit	21	Besprechen Sie die Generalisierbarkeit (externe Validität) der Studienergebnisse.	
**Zusätzliche Informationen**			
Finanzierung	22	Geben Sie an, wie die vorliegende Studie finanziert wurde, und erläutern Sie die Rolle der Geldgeber. Machen Sie diese Angaben gegebenenfalls auch für die Originalstudie, auf welcher der vorliegende Artikel basiert	
Zugänglichkeit des Protokolls, der Rohdaten und des Programmcodes		entfällt	RECORD 22.1: Die Autoren sollten darüber informieren, wie auf ergänzende Informationen wie Studienprotokoll, Rohdaten oder Programmcode zugegriffen werden kann.
